# Significant improvements in the olfactory sensitivity of bipolar I disorder patients during euthymia versus manic episodes: a longitudinal study

**DOI:** 10.3389/fpsyt.2024.1348895

**Published:** 2024-04-08

**Authors:** Xianlin Liu, Langjun Su, Yingying Li, Huiqian Yuan, Ao Zhao, Chunhong Yang, Chao Chen, Chunyang Li

**Affiliations:** Department of Psychiatry, Shunde WuZhongpei Memorial Hospital, Foshan, Guangdong, China

**Keywords:** bipolar I disorder, manic, olfactory sensitivity, olfactory identification, tumor necrosis factor-α, longitudinal study

## Abstract

**Introduction:**

Research has indicated that individuals diagnosed with bipolar disorder (BD) might experience alterations in their olfaction or levels of serum tumor necrosis factor-α (TNF-α), but no studies have investigated olfactory function and serum TNF-α in BD patients simultaneously. Moreover, there is a lack of existing research that compares the longitudinal olfactory function between individuals with manic and euthymic BD I.

**Methods:**

Patients with manic BD I (BDM, n=44) and healthy controls (HCs, n=32) were evaluated symptoms (measured via the Young Manic Rating Scale, YRMS), social function (measured via the Global Assessment Function, GAF), serum TNF-α, and olfactory function (via the Sniffin’ Sticks test) including olfactory sensitivity (OS) and olfactory identification (OI). The BDM patients were followed up to the remission period and re-evaluated again. We compared OS, OI and serum TNF-α in manic and euthymic patients with BD I and HCs. We examined the correlation between olfactory function and symptoms, social function, and serum TNF-α in patients with BD I.

**Results:**

The BDM patients exhibited significantly lower OS and OI compared to the HCs (Z = −2.235, P = 0.025; t = −6.005, P < 0.001), while a positive correlation was observed between OS and GAF score (r = 0.313, P = 0.039). The OS in the BD I remission group (n=25) exhibited significantly superior performance compared to the BDM group (t = −4.056, P < 0.001), and the same as that in the HCs (P = 0.503). The change in OS showed a positive correlation with the decrease in YMRS score (r = 0.445, P = 0.026), and a negative correlation with the course of disease (r = -0.594, P = 0.002). The TNF-α in BD I patients was significantly lower compared to HCs (P < 0.001), and not significantly correlated with olfactory function (all P > 0.05).

**Conclusion:**

The findings suggest that OS and OI are impaired in BDM patients, and the impaired OS in those patients can be recovered in the remission stage. OI may serve as a potential characteristic marker of BD. OS might be useful as an index for BDM treatment efficacy and prognosis.

## 1 Introduction

Bipolar disorder (BD) is primarily characterized by the occurrence of recurring and alternating episodes of mania or hypomania, as well as depression. According to the *Diagnostic and Statistical Manual of Mental Disorders, Fifth Edition (DSM-5)*, BD can be classified as bipolar I disorder (BD I) and bipolar II disorder (BD II). Compared with patients with BD II, who experience hypomania and depression, patients with BD I also experience manic episodes (1). However, there are no specific biological markers to inform psychiatric diagnoses of BD. Instead, the diagnostic criteria are defined by clinical history and psychiatric examination, so individuals with BD are often misdiagnosed with other conditions. Individuals with different mental disorders, and even different subtypes of BD, often need to be treated differently. For instance, the improper administration of antidepressant treatments to patients with depressive BD can induce mania (2). Therefore, identifying the biomarkers of BD holds significant importance in enhancing the precision and effectiveness of individual diagnosis and treatment approaches within the realm of clinical psychiatry.

Numerous research studies have provided evidence supporting the close association between emotion and the limbic system, comprising of the cingulate cortex, hippocampus, amygdala, and orbitofrontal cortex (3). Functional and anatomical irregularities within the limbic system have been detected through imaging examinations in individuals diagnosed with bipolar disorder (4–7). The limbic system also plays an important role in olfactory processing and the piriform cortex provides projections to the amygdala, hippocampus, and orbitofrontal cortex, serving as a crucial secondary olfactory region (8, 9). Accordingly, scholars have suggested that olfactory function might be implicated in BD (10, 11). Imaging studies exhibit noticeably reduced depth in their bilateral olfactory sulci compared to the healthy people (12). Sancaktar et al. (13) reported that compared with that in healthy controls (HCs), glucose metabolism in the olfactory bulb and amygdala was more active in patients with euthymic BD. This supports the hypothesis that emotional processing is primarily influenced by the olfactory bulb, and indicates that the olfactory bulb and amygdala are closely related to the pathogenesis of BD.

There is limited knowledge regarding the olfactory function in individuals diagnosed with BD, as the available information is scarce and inconsistent. Thus, the controversy surrounding the potential use of olfactory disorder as a biological marker for BD remains unresolved (14). Li et al. (15) reported that olfactory sensitivity (OS) in patients with BD was impaired only during the acute phase, and there was a negative association between OS impairment and the severity of clinical symptoms. In contrast, olfactory identification (OI) was impaired in patients in both the acute and euthymic phases of BD, so Li et al. proposed that OS might be a state indicator for BD, while OI could serve as a trait indicator of BD. However, Kazour et al. (16) carried out a cross-sectional study involving 33 patients with depressive BD, 30 patients with euthymic BD after depression, and 49 HCs. The results of their study indicated that there were no significant differences in the OS and OI between patients with depressive BD or euthymic BD, and HCs.

Inflammation is hypothesized to be a potential pathophysiological mechanism underlying BD (17), with cytokines playing a significant role in this process (18). As one of the pro-inflammatory cytokines, tumor necrosis factor-α (TNF-α) is generally associated with killing and inhibiting the activity of tumor cells. It also induces the production of other cytokines (19, 20). These cytokines, including TNF-α, are considered to be important factors related to the pathogenesis of BD, and may be appropriate targets for BD treatment (21). There is evidence that the pathophysiological mechanism underlying BD is associated with the modulation of neural plasticity and apoptosis, and that cytokines like TNF-α exert a significant influence on the pathogenesis of BD by influencing these processes (22).

Studies have indicated an elevation in peripheral blood TNF-α among BD patients. Van den Ameele et al. (23) revealed that in individuals diagnosed with BD, plasma TNF-α was increased in the acute phase and decreased in remission stage, such that it was consistent with that of HCs. Studies conducted by Munkholm et al. (24) and Modabbernia et al. (25) also reported a significant elevation in the concentration of TNF-α among BD patients when compared to HCs. However, a few studies suggested that TNF-α among BD patients did not increase (19, 20) or even decrease (26) in the acute phase.

In recent years, scholars have attempted to identify biomarkers to assist in the diagnosis of BD. Many cytokines, including TNF-α, have received increasing attention from researchers. These studies have indicated that TNF-α could potentially serve as a valuable biomarker for assisting in the diagnosis of BD. Research have indicated that patients with BD may exhibit abnormal olfactory function. Although there are inconsistent results, olfactory impairment has the potential to be a biological marker of bipolar disorder. In view of the fact that the sensitivity and specificity of olfactory function or peripheral TNF-α are not high enough when used alone for early diagnosis or efficacy evaluation of BD. This study simultaneously observed the changes of these two indicators in the acute phase and remission phase of BDM patients, and accumulated clinical reference data for future exploration of the adjuvant effect of TNF-α combined with olfactory function in the diagnosis and treatment of BD. A review published in 2023 suggested that inflammation may be involved in the pathophysiological mechanism of olfactory dysfunction in some psychiatric disorders (27). It can be inferred that cytokines, including TNF-α, may be associated with olfactory function in psychiatric disorders. Given that both impaired olfactory function and elevated peripheral TNF-α in BD may occur during the acute stage, but neither may show abnormal changes during the remission stage. It can be hypothesized that the pathophysiological mechanisms underlying these two phenomena are related to each other, resulting in the changes of olfactory function and peripheral TNF-α levels in BD patients.

Although previous studies basically grouped BDM patients with depressive BD and euthymic BD patients, limited studies have specifically examined the olfactory function of BDM individuals alone. A study by Kazour et al. (28) appears to be the first report on olfactory function in patients with BDM, but it was a cross-sectional comparison of participants in different groups instead of a longitudinal comparison of patients in the same group.

In summary, few studies have investigated olfactory function in patients with BDM alone. Furthermore, no studies have compared olfactory function longitudinally between individuals with manic and euthymic BD I, or analyzed the relationship between serum TNF-α level and olfactory dysfunction among patients with BD. The objective of this study was to provide a new index to facilitate the early detection of BD I or to assess treatment outcomes, and to provide new clinical evidence regarding the pathophysiological mechanisms of BD.

We postulated that patients with manic BD I (BDM) may exhibit impaired olfactory function and elevated serum TNF-α, but that these changes would return to normal levels during the remission period. Furthermore, we hypothesized that there might be a potential association between olfactory function and serum TNF-α levels among these individuals.

## 2 Methods

### 2.1 Participants

The study involved individuals diagnosed with BDM who were hospitalized at Shunde WuZhongpei Memorial Hospital, Foshan City, Guangdong, China, from July 2022 to December 2022. The patients were followed up and evaluated during hospitalization or as outpatients when they were in the remission period. At the same time, HCs were recruited from the healthy hospital staff who had matched social and demographic characteristics as the case group. The patients in the BDM group were diagnosed with BDM according to the *DSM-5*, had no other mental disorders, and received a Young Mania Rating Scale (YMRS) score ≥20 and a Hamilton Rating Scale for Depression (HAMD) score <12. The BD I remission (BDR) group were the same BDM patients, assessed during the follow-up period. They were re-evaluated when there had been no obvious clinical symptoms for at least one month, and received a YMRS score ≤7 and HAMD score ≤8. The HCs also had no mental illness, and had a YMRS score ≤7 and HAMD score ≤8.

All participants were of Chinese Han nationality, aged 18–60 years, and had attended primary school education or above. They had no history of head or neck trauma, nasal surgery, neurological diseases (epilepsy, stroke), intellectual disability, or any diseases that could impact olfactory function (like nasal polyps, common colds, or allergies). Individuals with a prior record of alcohol or substance abuse or dependence within the last year, as well as those unable to complete the olfactory examination or scale evaluation, were not included in the study.

**Figure 1:**
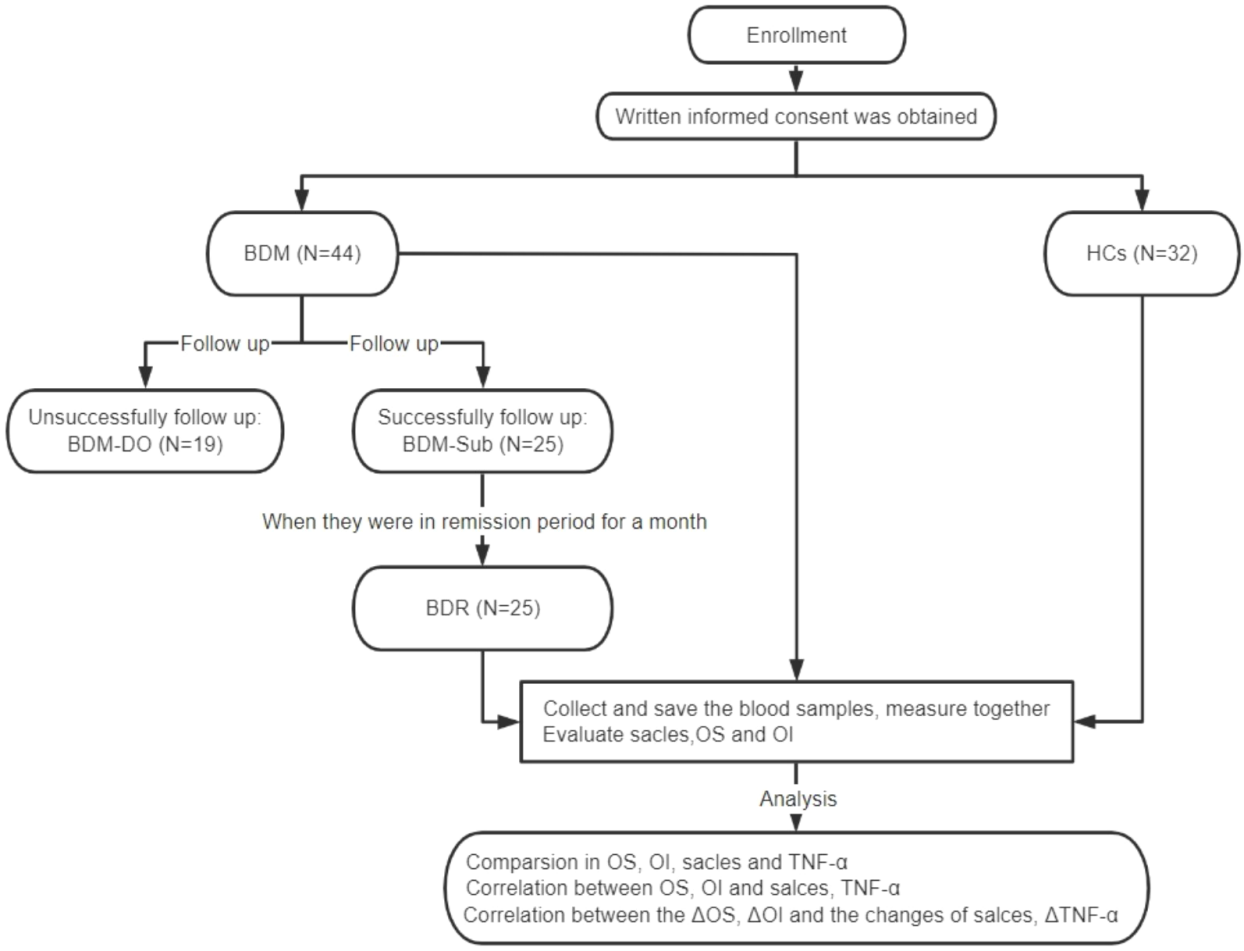
CONSORT diagram for assessments of olfactory function and serum TNF-α levels. BDM, patients with manic BD I; HCs, healthy controls; BDM-DO, patients with manic BD I who dropped out during the follow-up; BDM-Sub, sub group of BDM, patients with manic BD I who could be followed up to the remission period; BDR, patients with manic BD I who were followed up to the remission period; OS, olfactory sensitivity; OI, olfactory identification; TNF-α, tumor necrosis factor-α; ΔOS, change in olfactory sensitivity; ΔOI, change in olfactory identification; ΔTNF-α, change in tumor necrosis factor-α levels.

The experimental flow and study steps are shown in **Figure 1**.

This study was approved by the Medical Ethics Committee of Shunde WuZhongpei Memorial Hospital, Foshan City, Guangdong, China, and conducted in accordance with the principles outlined in the Declaration of Helsinki. Participants or their family members were duly informed and consented in writing. Our study strictly adhered to the principle of voluntary participation, allowing participants to withdraw at any given time.

### 2.2 Measures

The YMRS: This is primarily employed for the assessment of manic symptoms; the score range is 0–60. Generally, YMRS scores of 0–5 suggest the absence of evident manic symptoms, scores ranging from 6 to 10 indicate the presence of manic symptoms, and score more than 22 indicates severe manic symptoms.

HMAD: This is one of the most frequently utilized scales employed to assess depression in clinical practice. In our study, we used the 24-item version of the HAMD (HAMD-24). A total HAMD-24 score >20 reflects mild or moderate depressive symptoms, while HAMD-24 <8 indicates no depressive symptoms.

Global Assessment Function (GAF): This is employed for the assessment of the psychological, social, and professional functions of the participants. GAF has only one statistic, that is, the total score (1–100 points). A higher score indicates a milder disease state. For each participant, we evaluated GAF, HAMD and YMRS in person, with two psychiatrists who were trained in the use of the scales as evaluators.

Sniffin’ Sticks test (SST): The SST serves as a tool for measuring quantitative olfactory abilities that can test OS and OI respectively. The OS test comprised a total of 16 n-butanol olfactory felt-tip pens and corresponding blank pens. The pen exhibited a 4% concentration of n-butanol, which was the highest among all the pens, and the other pens contained varying concentrations, corresponding to concentration levels 1–16, respectively. Before the OS test, the No. 1 n-butanol pen was given to the subject to familiarize them with the test odor. Each group was tested with one n-butanol olfactory pen and two blank pens. The determination of OS was achieved through the utilization of a single staircase method. The triplets were presented to the subject successively, until they accurately identified the scent in two consecutive trials, leading to a reversal of the staircase. The threshold estimate was determined by calculating the geometric mean of the last four points where reversals occurred out of a total of seven reversals. A higher OS score corresponded with greater OS. The OI examination comprised a collection of 16 distinct olfactory sticks. The participants were requested to recognize the 16 odors and choose the correct answer for each stick from four options provided. Each accurate response was recorded as 1 point, while an inaccurate response received no points. The cumulative score amounted to 16 points. A higher OI score corresponded with greater OI (29).

Serum TNF-α: The TNF-α ELISA kit (Shanghai Renjie Biotechnology Co., Ltd.) was used to detect sample concentrations via a double-antibody sandwich ELISA. A fasting venous blood sample of 5ml was collected from the subjects in the morning and centrifuged after natural coagulation at room temperature. The centrifugation process lasted approximately 20 minutes at a speed of 2000-3000 revolutions per minute. Carefully collect the supernatant and store it in the refrigerator at -40°C for measurement. Two dedicated technicians belonging to our team members carried out the experiment in the laboratory of Shunde WuZhongpei Memorial Hospital, Foshan City, Guangdong, China, according to the manufacturer’s instructions of the TNF-α ELISA kit.

### 2.3 Statistics

SPSS 25.0 software (SPSS Inc, Chicago, IL, USA) was utilized for conducting statistical analyses. The number of cases and constituent ratios were used to present the qualitative data, while the chi-square test was employed for analysis. The normality of the quantitative data was tested, and the measurement data that followed a normal distribution are presented as the mean ± standard deviation. Otherwise, they are shown as the median values (25th percentile, 75th percentile) [M (P_25_, P_75_)]. Considering the sample size of our study, we used the Shapiro-Wilk test to determine normality. A comparison between the two groups of data with a normal distribution was conducted using either an independent sample t-test or paired sample t-test. For non-normally distributed data, the comparison was performed using either the Mann-Whitney U test or Wilcoxon signed rank sum test. The relationships were analyzed via Pearson’s correlation or Spearman’s correlation. The significance level for the tests was set at α = 0.05, and both two-tailed tests were performed.

## 3 Results

### 3.1 Comparison between the BDM and HC groups

**Figure 2:**
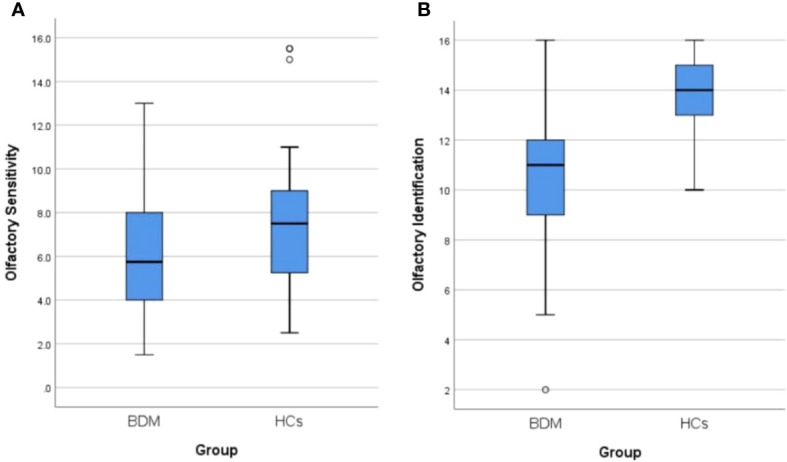
**(A)** Comparison of olfactory sensitivity between BDM and HCs. **(B)** Comparison of olfactory identification between BDM and HCs. BDM, patients with manic BD I; HCs, healthy controls.

A total of 44 patients with BDM and 32 HCs were included in the study. There was no notable distinctions in age, gender, smoking habits, or education background between the two groups (all P > 0.05). The OS and OI in the two groups were compared via the Mann-Whitney U test and independent sample t-test respectively, and we found significant differences in both OS and OI between the two groups (Z = −2.235, P = 0.025 and t = −6.005, P < 0.001, respectively). As shown in **Table 1**, which includes a box chart showing the OS and OI, the BDM group exhibited poorer performance in both the OS and OI compared to the HCs (see **Figures 2A, B**). We observed a significant difference in serum TNF-α levels between the two groups (Z = −4.650, P < 0.001), suggesting that the BDM group exhibited reduced serum TNF- α compared to the HCs.

**Table 1:** Sociodemographics, clinical characteristics, olfactory function, and TNF-α levels in individuals with BDM and HCs.

Variable	BDM (N=44)	HCs (N=32)	t/χ2/Z	P
Sociodemographics				
** Gender, Male (%)** ** Age (years)** ** Smoking, N (%)** ** Years of education**	25 (56.8) 36.2 ± 11.8 15 (34.1) 11.5 (9.0, 14.8)	17 (53.1) 36.8 ± 8.4 7 (21.9) 15.0 (9.5, 15.0)	0.102 -0.253 1.344 -1.889	0.749 0.801 0.246 0.059
Clinical characteristics				
** Course of disease (years)** ** YMRS** ** GAF**	10.0 (6.0, 15.5) 27.5 (24.0, 33.0) 42.0 (32.0, 45.8)	/ 0.0 (0.0, 2.0) 94.0 (92.0, 95.8)	/ / /	/ / /
Medications^b^				
** Antipsychotics, N (%)** ** Risperidone** ** Olanzapine** ** Quetiapine** ** Mood stabilizers, N (%)** ** Lithium** ** Valproate** ** Benzodiazepines, N (%)**	39 (88.6) 12 (27.3) 10 (22.7) 13 (29.5) 41 (93.2) 26 (59.1) 31 (70.5) 28 (63.6)	/ / / / / / / /		
Olfactory function				
** OS**	5.8 (4.0, 8.0)	7.5 (5.1, 9.0)	-2.235	0.025^a^
** OI**	10.6 ± 2.8	13.6 ± 1.5	-6.005	< 0.001^a^
Serum TNF-α (pg/ml)	16.8 (15.2, 18.2)	22.0 (20.4, 23.4)	-4.650	< 0.001^a^

BDM, patients with manic BD I; HCs, healthy controls; YMRS, Young Mania Rating Scale; GAF, Global Assessment Function; OS, olfactory sensitivity; OI, olfactory identification; TNF-α, tumor necrosis factor-α.

^a^Values are statistically significant; ^b^Patients may have received more than 1 mediation type.

### 3.2 Comparison between the euthymic BD and HC groups

The BD symptoms, olfactory function, and TNF-α were followed up in the BDM group. At the end of the study in December 2022, a total of 25 patients were in remission and had successfully completed the follow-up. The follow-up completion rate was 56.82% (before completion of the follow-up, the subgroup was called the “BDM subgroup (BDM-sub)”; after completion of the follow-up, the group was called the “BD I remission group (BDR)”). There were no notable disparities in age, gender, smoking habits, or education background between these 25 patients and the 32 HCs (all P > 0.05). In the BDR patients, the OI was worse than that in the HCs (Z = −4.039, P < 0.001), but there were no notable disparities in OS between the two groups (Z = −0.669, P = 0.503). Serum TNF-α levels in the BDR patients were found to be significantly decreased compared to those observed in the HCs (Z = −4.535, P < 0.001). See **Table 2**.

**Table 2:** Sociodemographics, clinical characteristics, olfactory function, and TNF-α levels in BDR patients and HCs.

Variable	BDR (N=25)	HCs (N=32)	t/χ2/Z	P
Sociodemographics				
** Gender, Male (%)** ** Age (years)** ** Smoking, N (%)** ** Years of education**	13 (52.0) 32.4 ± 8.6 11 (44.0) 12.0 (10.5, 15.0)	17 (53.1) 36.8 ± 8.4 7 (21.9) 15.0 (9.5, 15.0)	0.007 -1.948 3.180 -1.200	0.933 0.056 0.075 0.230
Clinical characteristics				
** Course of disease (years)** ** YMRS** ** GAF**	9.4 ± 6.6 4.0 (0.5, 6.5) 81.0 (75.0, 85.5)	/ 0.0 (0.0, 2.0) 94.0 (92.0, 95.8)	/ / /	/ / /
Medications^b^				
** Antipsychotics, N (%)** ** Risperidone** ** Olanzapine** ** Quetiapine** ** Mood stabilizers, N (%)** ** Lithium** ** Valproate** ** Benzodiazepines, N (%)**	25 (100.0) 10 (40.0) 4 (16.0) 9 (36.0) 25 (100.0) 21 (84.0) 20 (80.0) 13 (52.0)	/ / / / / / / /		
Olfactory function				
** OS**	7.5 (6.3, 9.3)	7.5 (5.1, 9.0)	-0.669	0.503
** OI**	12.0 (11.0, 13.0)	14.0 (13.0, 15.0)	-4.039	< 0.001^a^
Serum TNF-α (pg/ml)	16.7 (15.5, 17.5)	22.0 (20.4, 23.4)	-4.535	< 0.001^a^

BDR, patients with manic BD I who were followed up to the remission period; HCs, healthy controls; YMRS, Young Mania Rating Scale; GAF, Global Assessment Function; OS, olfactory sensitivity; OI, olfactory identification; TNF-α, tumor necrosis factor-α.

^a^Values are statistically significant; ^b^Patients may have received more than 1 mediation type.

### 3.3 Comparison between the BDM and euthymic BD groups

#### 3.3.1 Comparison between the BDM subgroup and the BDM drop-out subgroup

There were 19 patients who failed to complete the follow-up assessment at the end of the study (called the “BDM drop-out subgroup (BDM-DO)”), of which 5 patients were still in the manic state at the end of the follow-up period. Another 14 patients were discharged after hospitalization, but they were not evaluated again after discharge because of personal reasons. There were significant differences in age, YMRS score, GAF score, and medication type between the two subgroups, but no significant differences in the other items (P > 0.05). This suggests that clinical symptoms and impaired social function in the BDM-DO may have been more serious than those in BDM-sub, which may be related to the fact that some patients in the BDM-DO did not achieve remission at the follow-up endpoint. Although there were differences in age, clinical symptoms, and social function between the BDM-sub and BDM-DO, there were no disparities in gender, smoking, education, or course of disease, and there were no disparities in OS, OI, or serum TNF-α at the time of inclusion between the two subgroups. See **Table 3**.

**Table 3:** Sociodemographics, clinical characteristics, medications, olfactory function, and TNF-α levels in the BDM-Sub versus BDM-DO.

Variable	BDM-Sub (N=25)	BDM-DO (N=19)	t/χ2/Z	P
Sociodemographics				
** Gender, Male (%)** ** Age (years)** ** Smoking, N (%)** ** Years of education**	13 (52.0) 32.4 ± 8.6 11 (44.0) 12.0 (10.5, 15.0)	12 (63.2) 41.3 ± 13.7 4 (21.1) 7.0 (9.0, 15.0)	0.548 ^b^ -2.482 2.530 -1.157	0.459 0.019^a^ 0.112 0.247
Clinical characteristics				
** Course of disease (years)** ** YMRS** ** GAF**	8.0 (5.0, 13.0) 25 (22.5, 30.5) 43.0 ± 11.2	10.0 (6.0, 21.0) 31.0 (26.0, 35.0) 36.3 ± 9.8	-1.511 -2.445 2.084	0.131 0.014 ^a^ -0.043 ^a^
Medications^b^				
**Medication type** ** Antipsychotics, N (%)** ** Risperidone** ** Olanzapine** ** Quetiapine** ** Mood stabilizers, N (%)** ** Lithium** ** Valproate** ** Benzodiazepines, N (%)**	4 (3, 4) 25 (100.0) 10 (40.0) 4 (16.0) 9 (36.0) 25 (100.0) 16 (64.0) 22 (88.0) 13 (52.0)	3 (3, 4) 17 (89.5) 3 (15.8) 7 (36.9) 5 (26.3) 16 (84.2) 10 (52.6) 9 (47.4) 10 (52.6)	2.552 / / / / / / / /	0.011^a^ / / / / / / / /
Olfactory function				
** OS**	5.8 ± 2.6	6.4 ± 2.4	-0.820	0.417
** OI**	11.0 (9.0, 13.0)	10.0 (8.0, 12.0)	-0.526	0.599
Serum TNF-α (pg/ml)	17.3 (15.9, 18.4)	16.6 (14.7, 18.2)	-0.735	0.463

BDM-Sub, sub group of BDM, patients with manic BD I could be followed up to the remission period; BDM-DO, patients with manic BD I who dropped out from the follow-up; YMRS, Young Mania Rating Scale; GAF, Global Assessment Function; OS, olfaction sensitivity; OI, olfaction identification; TNF-α, tumor necrosis factor-α.

^a^Values are statistically significant; ^b^Patients may have received more than 1 mediation type.

#### 3.3.2 Changes in olfactory function and TNF-α in the BDM subgroup versus euthymic BD group

According to a paired sample t-test, the impaired OS in patients with BDM-sub was significantly improved during remission (t = −4.056, P < 0.001). However, there was no notable disparities in OI or serum TNF-α between the two groups, as determined by the Wilcoxon symbolic rank sum test. See **Table 4**.

**Table 4:** Olfactory function and TNF-α levels in the BDM-Sub and BDR groups.

Variable	BDM-Sub (N=25)	BDR (N=25)	t/Z	P
Olfactory function				
** OS**	5.8 ± 2.6	8.0 ± 2.3	-4.056	< 0.001^a^
** OI**	11.0 (9.0, 13.0)	12.0 (11.0, 13.0)	-0.945	0.345
TNF-α (pg/ml)	17.3 (15.9, 18.4)	16.7 (15.5, 17.5)	-0.901	0.367

BDM-Sub, sub group of BDM, patients with manic BD I who could be followed up to the remission period; BDR, patients with manic BD I who were followed up to the remission period; OS, olfactory sensitivity; OI, olfactory identification; TNF-α, tumor necrosis factor-α.

a Values are statistically significant.

### 3.4 Correlation analysis in the BDM and euthymic BD groups

The correlation analysis (Pearson/Spearman) between the OS or OI and age, course of disease, years of education, YMRS score, GAF score, and serum TNF-α in the BDM group showed a positive correlation between the OS and GAF score in the BDM group (r = 0.313, P = 0.039). Furthermore, the analysis of Pearson’s correlation revealed a positive association between OS and OI in the BDM group (r = 0.380, P = 0.011), indicating that the OS and OI were impaired synchronously in patients with BDM. However, there was no significant correlation found between age, course of disease, years of education, YMRS score, GAF score, or TNF-α levels with either the OS or OI in the BDR group (all P > 0.05).

Correlations (Pearson/Spearman) were analyzed respectively between the changes in OS or OI (ΔOS or ΔOI, respectively) in BDR patients and other indexes, including age, course of disease, years of education, decrease in YMRS score (ΔYMRS, reflecting the recovery of manic symptoms), change in GAF score (ΔGAF, reflecting the recovery of manic symptoms), and change in TNF-α levels (ΔTNF-α). The results showed that ΔOS in BDR patients was negatively correlated with the course of the disease (r = -0.594, P = 0.002) and positively correlated with the decrease in YMRS score (r = 0.445, P = 0.026). See **Table 5**.

**Table 5:** Relationships between change in olfactory function and other indexes in BDR patients.

Variable	ΔOS		ΔOI	
	r	P	r	P
Sociodemographics				
** Age (years)** ** Years of education**	-0.386 -0.052	0.057 0.806	-0.284 -0.129	0.168 0.537
Clinical characteristics				
** Course of disease (years)** ** ΔYMRS** ** ΔGAF**	-0.594 0.445 0.179	0.002^a^ 0.026^a^ 0.391	-0.111 -0.145 -0.126	0.597 0.489 0.549
ΔTNF-α (pg/ml)	-0.078	0.710	-0.172	0.412

BDR, patients with manic BD I followed up to the remission period; ΔYMRS, decrease in Young Mania Rating Scale score; ΔGAF, change in Global Assessment Function score; ΔOS, change in olfactory sensitivity; ΔOI, change in olfactory identification; ΔTNF-α, change in tumor necrosis factor-α levels.

a Values are statistically significant.

## 4 Discussion

### 4.1 Olfactory sensitivity

In this study, we observed that the OS of BDM individuals was significantly lower compared to that of the HCs, and that impaired OS could be recovered in the remission phase. No notable distinction was observed between individuals with euthymic BD and HCs. With regard to OS, the results of Kazour et al. (28) and Li et al. (15) were not consistent. Li et al. (15) revealed that the OS in patients with BDM was comparatively reduced compared to the HCs, which is aligning with our study. Kazour et al. (28) showed that the OS in patients with BDM was comparable to that of HCs, which contradicts the findings of our own research. A potential explanation could be the disparity in gender distribution within the patient group: in Kazour^’^s study, there were fewer men (44.4%), while in Li^’^s study and our study, the proportion of men with bipolar mania was higher, 58.6% and 56.8%, respectively. Kamath et al. (30) pointed out that in a test of OS in patients with acute mood disorder, olfactory function may be different in patients with acute mood disorder because of gender-bcased differences.

Li et al. (15) and Kazour et al. (16, 28) similarly reported that there was no significant disparity in OS among euthymic BD patients compared to HCs. The results of our study align well with these previous investigations. In addition, Hardy et al. (10) found that there was no obvious difference in OS between 20 patients with stable BD and 44 HCs, which seems to support the results of this study. However, the result of Hardy’s study should be interpreted carefully because of different definitions and criteria for the stable condition or remission among different studies. In addition to the potential influence of gender differences, Kazour et al. (28) pointed out in the summary of their report that the results of longitudinal studies on olfactory function in the same group of patients should be more accurate than that of cross-sectional studies.

### 4.2 Olfactory identification

In our study, OI was obviously impaired in BDM patients when compared to that observed in HCs, and this impairment was present in euthymic patients. Kazour et al. (28) and Li et al. (15) revealed that OI was impaired in patients with BDM, which is in line with our findings.

During the remission stage, Lahera et al. (11) found that 39 individuals with euthymic BD had impaired OI compared with HCs. The results reported by Kazour et al. (28) and Li et al. (15) are similar to those of Lahera et al., and all are consistent with the results of the current study. Furthermore, a research conducted by Kazour et al. (16) demonstrated that 33 patients with euthymic BD had poorer OI for pleasant odors compared with HCs, although no overall impairment in OI was found when comparing these patients with 49 HCs. Kamath et al. (31) conducted a study that included 43 BD I patients (of which 13 with psychotic symptoms) and 72 HCs, and showed that BD I patients had impaired OI in comparison to HCs. Although their study did not specify the disease status of their patients, the results were consistent with our study.

### 4.3 Correlation between olfactory function and various indexes (except TNF-α)

We examined the association between olfactory function, manic symptoms, and social function among BDM patients, and discovered a positive correlation between OS and social function. A lower OS was associated with poorer social function. A change in OS demonstrated a positive association with a reduction in YMRS score (reflecting the improvement in manic symptoms) and exhibited a negative association with the course of disease, indicating that the better the recovery of manic symptoms, the better the recovery of impaired OS, and the longer the course of disease, the worse the recovery of impaired OS.

Hardy et al. (10) reported a positive association between the OS with social function in BD patients, which was in accordance with the findings of this study. Furthermore, the occurrence of anosmia was found to have an adverse effect on patient daily life, social relationships, and the ability to work (32). Although we did not observed significant association between OI and social function among BD patients in this investigation, Cumming et al. (33) previously reported that OI and social function were correlated in this patient group. This discrepancy may be related to the use of different tools in different studies. To evaluate social function, Cumming et al. used the Zigler Social Competence Scale, which considers education background, professional standing, work experience, and marital situation. In our study, we used the GAF to assess social function with a single subjective score, which may be less accurate.

As for the relationship between OS and manic symptoms, Hardy et al. (10) found that these variables were correlated, such that increased severity of manic symptoms was associated with decreased OS. Li et al. (15) also reported a negative association between the YMRS score and OS in patients with BD. The above results are similar to those of our study. Although we failed to establish a correlation between OS and manic symptoms during BDM or euthymic BD, we found that the degree of OS recovery after BDM remission was positively correlated with the degree of manic symptom recovery (the reduction in YMRS score). Future studies should expand the sample size to further clarify the correlation between olfactory dysfunction and manic symptoms in patients with BD.

In addition, we found that the recovery of OS after remission in patients with BDM exhibited a negative correlation with the course of the disease. A possible explanation is that most patients with BD have a chronic course of disease and tend to have recurrent episodes (34). The chronic nature of the disease leads to increased structural and functional abnormalities within the brain areas shared by BD and olfaction (e.g., orbitofrontal cortex and hippocampus), slowing the OS recovery process.

### 4.4 Relationship between TNF-α and olfactory function

In this study, it was observed that the levels of serum TNF-α were comparatively reduced in BD patients when compared to those in HCs during both the manic phase and the subsequent remission stage. Most prior investigations have indicated that peripheral TNF-α among BD patients is either elevated or comparable to those observed in HCs. Luo et al. (35) and Solmi et al. (36) found that the serum TNF-α levels were markedly elevated in individuals with BD during the acute phase compared to those in HCs. The latter study also revealed that there was no statistically significant difference in the TNF-α level between patients with euthymic BD and HCs. Studies of peripheral TNF-α have also shown elevated levels in BD I patients. Wang et al. (37) and Chen et al. (38) respectively observed that serum TNF-α among patients with BD I was significantly higher than that in HCs.

Pantović-Stefanović et al. (26) carried out a longitudinal follow-up study involving 83 patients with acute BD I, and showed that the levels of serum TNF-α in the patients who successfully achieved remission after 10 weeks of treatment were significantly lower compared to those of HCs in both the acute phase and remission stage. The findings of the present study are in line with these results, which may be attributed to the inherent characteristics of TNF-α. The pro-inflammatory cytokine TNF-α is implicated in the initial inflammatory process of BD, while also exhibiting anti-inflammatory properties and contributing to the subsequent anti-inflammatory response (39). Figiel (40) pointed out that TNF-α might have a protective effect on the brain. Therefore, the observed decrease in TNF-α levels among BD patients in current study might be explained by the relationship between the decline in TNF-α levels and the chronic course of BD. Although our study did not discover any association between serum TNF-α levels and the course of disease among BD patients in our study, Pantović-Stefanović et al. (26) observed a negative correlation between untreated course of disease and serum TNF-α levels in BD patients.

We observed olfactory dysfunction in patients with manic and euthymic BD, as well as reduced serum TNF-α levels, but no association between the olfactory dysfunction and serum TNF-α levels. At present, there is a lack of research indicating any connection between TNF-α and olfactory function in patients with BDM or euthymic BD. These two pathophysiological mechanisms of BD are relatively complex with many influencing factors, and may be independent from one another. Thus, it may not be possible to describe the relationship between them using a simple linear correlation.

## 5 Limitations

The study has several limitations that should be acknowledged. Firstly, the sample size was relatively small, and a large number of patients dropped out during the follow-up period. Secondly, the subjective nature of the olfactory function measurement method employed in this study could have potentially influenced the outcomes. In the future, we hope to combine objective olfactory detection methods. Thirdly, all the patients included in this study were under medication, which was not controlled. Thus, we could not exclude the influence of different therapeutic medications on olfactory function and serum TNF-α in patients with BD.

## 6 Conclusions

In patients with BDM, both OS and OI showed synchronous impairment when compared to those in the HCs, and OS was positively correlated with GAF score. Impaired OS in patients with BDM can be recovered in the remission stage, and the degree of recovery of OS exhibits a positive correlation with the degree of recovery of manic symptoms. We observed no statistically significant disparity in OS between patients with euthymic BD I and HCs, suggesting that OS could be a new marker for treatment efficacy and BDM prognosis. The OI in patients with BDM was still significantly impaired even when manic symptoms were recovered, suggesting that OI could potentially serve as a trait marker in bipolar manic patients. We observed no significant correlation between olfactory ability and peripheral TNF-α in BDM patients, suggesting that the pathophysiological mechanism of olfactory function and TNF-α in BD may be independent.

## Data availability statement

The original contributions presented in the study are included in the article/supplementary material. Ffurther inquiries can be directed to the corresponding author.

## Ethics statement

The studies involving humans were approved by the Medical Ethics Committee of Shunde WuZhongpei Memorial Hospital, Foshan City, Guangdong, China. The studies were conducted in accordance with the local legislation and institutional requirements. The participants provided their written informed consent to participate in this study. Written informed consent was obtained from the individual(s) for the publication of any potentially identifiable images or data included in this article.

## Author contributions

XL: Writing – original draft, Investigation, Formal analysis, Data curation, Methodology, Conceptualization. LS: Investigation, Data curation, Writing – original draft. YL: Investigation, Data curation, Writing – original draft. HY: Investigation, Data curation, Writing – original draft. AZ: Data curation, Writing – original draft. CY: Investigation, Data curation, Writing – original draft. CC: Writing – review & editing, Supervision. CL: Writing – review & editing, Supervision, Methodology, Conceptualization.
